# The Profile of Post-translational Modifications of Histone H1 in Chromatin of Mouse Embryonic Stem Cells

**DOI:** 10.32607/20758251-2019-11-2-82-91

**Published:** 2019

**Authors:** T. Yu. Starkova, T. O. Artamonova, V. V. Ermakova, E. V. Chikhirzhina, M. A. Khodorkovskii, A. N. Tomilin

**Affiliations:** Institute of Cytology of the Russian Academy of Sciences, Laboratory of Molecular Biology of Stem Cells, Tikhoretsky Ave. 4, St. Petersburg, 194064, Russia; Peter the Great St.Petersburg Polytechnic University, Politekhnicheskaya Str. 29, St. Petersburg, 195251 , Russia; Saint Petersburg State University, 13B Universitetskaya Emb., St. Petersburg, 199034, Russia

**Keywords:** mouse embryonic stem cells, linker histone H1, post-translational modifications, 2-D electrophoresis, MALDI mass spectrometry

## Abstract

Linker histone H1 is one of the main chromatin proteins which plays an important role in organizing eukaryotic DNA into a compact structure. There is data indicating that cell type-specific post-translational modifications of H1 modulate chromatin activity. Here, we compared histone H1 variants from NIH/3T3, mouse embryonic fibroblasts (MEFs), and mouse embryonic stem (ES) cells using matrix-assisted laser desorption/ ionization Fourier transform ion cyclotron resonance mass spectrometry (MALDI-FT-ICR-MS). We found significant differences in the nature and positions of the post-translational modifications (PTMs) of H1.3-H1.5 variants in ES cells compared to differentiated cells. For instance, methylation of K75 in the H1.2-1.4 variants; methylation of K108, K148, K151, K152 K154, K155, K160, K161, K179, and K185 in H1.1, as well as of K168 in H1.2; phosphorylation of S129, T146, T149, S159, S163, and S180 in H1.1, T180 in H1.2, and T155 in H1.3 were identified exclusively in ES cells. The H1.0 and H1.2 variants in ES cells were characterized by an enhanced acetylation and overall reduced expression levels. Most of the acetylation sites of the H1.0 and H1.2 variants from ES cells were located within their C-terminal tails known to be involved in the stabilization of the condensed chromatin. These data may be used for further studies aimed at analyzing the functional role played by the revealed histone H1 PTMs in the self-renewal and differentiation of pluripotent stem cells.

## INTRODUCTION

Chromatin architectural proteins include structural proteins, such as histone H1, which are devoid of enzymatic activity, bind nucleosomes without apparent DNA sequence specificity, and change the local and global architecture of chromatin [1-8]. Proteins belonging to the human and mouse histone H1 families include seven somatic subtypes (H1.0 through H1.5, and H1X), three testis-specific variants (H1t, H1T2m, and HILS1), and one variant restricted to oocytes (H1oo) [9-13]. The H1 variants have different evolutionary stability, euchromatin/heterochromatin distribution, and chromatin-binding affinity, which may be a result of post-translational modifications [14-17].

Over the past few decades, chromatin of ES cells and iPS cells has been the focus of extensive research because of the tremendous potential of these cells in biomedicine. Chromatin of these cells has some unique structural features that distinguish it from chromatin of differentiated cells [17-18]. In particular, heterochromatin of ES cells appears to be more relaxed due to a reduced expression of H1 proteins [19] and PTMs of nuclear proteins [18-20], leading to globally increased transcription. In this study, we compared PTMs of the H1 variants from mouse–differentiated and ES cells. We report on novel ES cell-specific PTMs of H1 and discuss the potential impact of these PTMs on H1 functions and the structure of chromatin in ES cells.

## MATERIALS AND METHODS

**Ethics statement**

All animal procedures were performed according to the Guidelines for the Humane Use of Laboratory Animals, with standards complying with those approved by the American Physiological Society. Mouse experiments were conducted strictly in agreement with the animal protection legislation acts of the Russian Federation and were approved by the Institute’s Ethics Board as complying with the requirements for humane use of laboratory animals.

Mouse embryonic fibroblasts (MEFs) were isolated using animals after natural mating, which were sacrificed using the UK Home Office “Schedule 1” procedure requiring no specific ethical approval. The E14Tg2A cell culture was procured from BayGenomics. The NIH/3T3 cells were obtained from the Russian Cell Culture Collection (Institute of Cytology, St. Petersburg, Russia), where they were authenticated by STR DNA profiling analysis.

**Mouse cell cultures**

NIH/3T3 cells obtained from ATCC and mouse embryonic fibroblasts (MEFs) prepared from mid-gestation mouse embryos [21, 22] were cultured in DMEM supplemented with 10% fetal bovine serum, L-glutamine, and 1% penicillin/streptomycin. Mouse ES cells (line E14Tg2A, BayGenomics) were cultured on gelatin-coated dishes in DMEM/F12 supplemented with 15% fetal bovine serum, 1% penicillin/streptomycin, L-glutamine, NEAA, and leukemia inhibitory factor (LIF). The cells were washed with PBS (pH 7.5), harvested with 0.05% trypsin (10 min at 37°C), and collected by centrifugation at 2,000 g for 5 min. Pellets were frozen in liquid nitrogen and stored at -70°C. To prepare the H1 samples for subsequent analysis, cells were collected from six plates (d = 10 cm).

**Histone H1 variant extraction and separation**

To preserve as much of the PTMs as possible, H1 proteins were extracted directly from frozen pellets, avoiding nucleus isolation, according to the previously described procedure [7]. The H1 variants were separated by 2-D electrophoresis as described previously [7, 8].

**Digestion and MALDI-FT-ICR-MS analysis**

Following 2-D electrophoresis, gel fragments containing nuclear proteins were cut out, minced, and treated as described previously [7]. Biological samples were analyzed in two biological and two or three analytical replicates. The mass spectra were recorded and analyzed as described previously [7].

## RESULTS

**Fig. 1:**
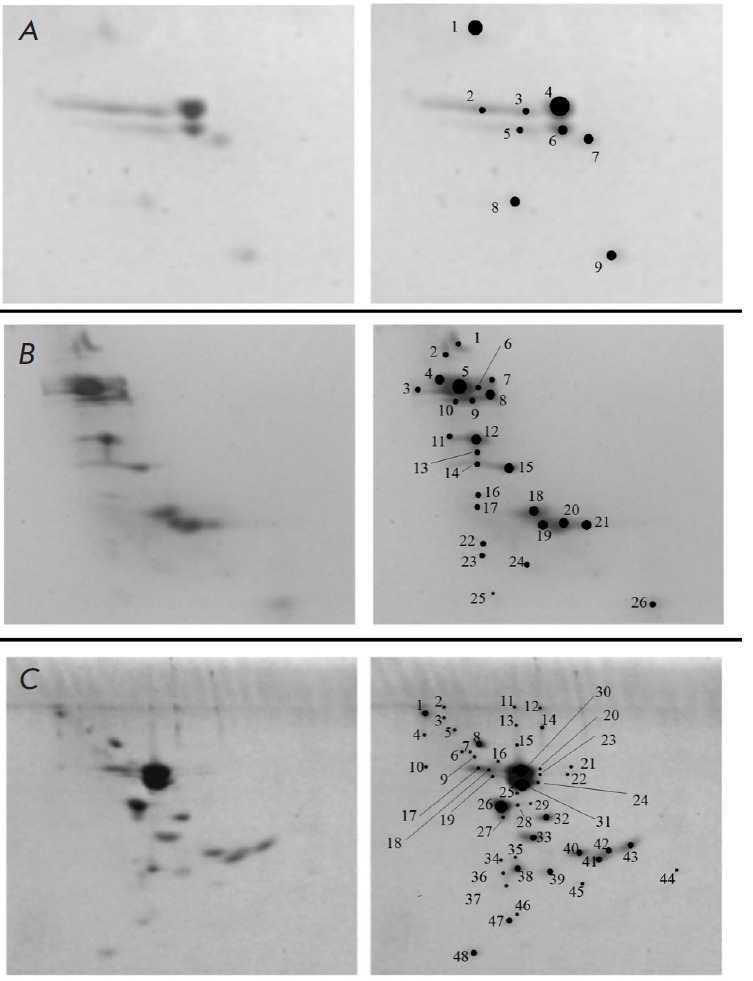
Two-dimensional gel electrophoresis of H1- enriched extracts from NIH/3T3 cells (A), MEFs (B), and ES cells (C). H1 variants were identified in five fractions (marked 2–4, 6–7 in A), seven fractions (marked 4–10 in B), and eight fractions (marked 15–18, 20–21, 30–31 in C) for NIH/3T3, MEFs, and ES cells, respectively. The remaining fractions were attributed to the HMGB and HMGN of High-Mobility Group family proteins and other nuclear proteins (Table S1 [25])

**Fig. 2:**
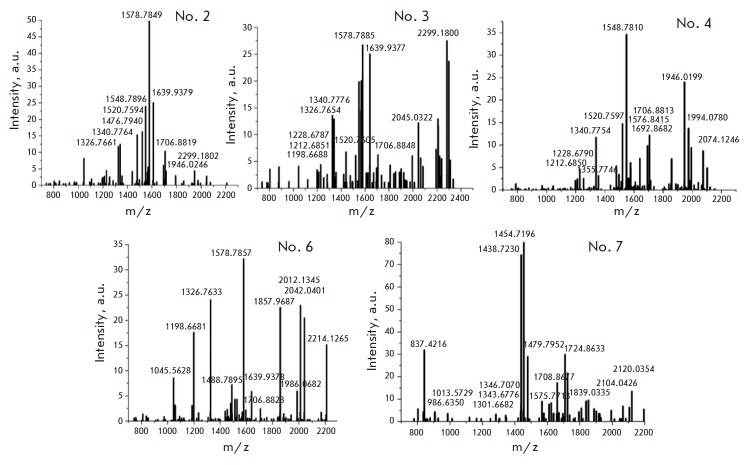
Mass spectrum of the 2D NIH/3T3 H1 zones

**Fig. 3:**
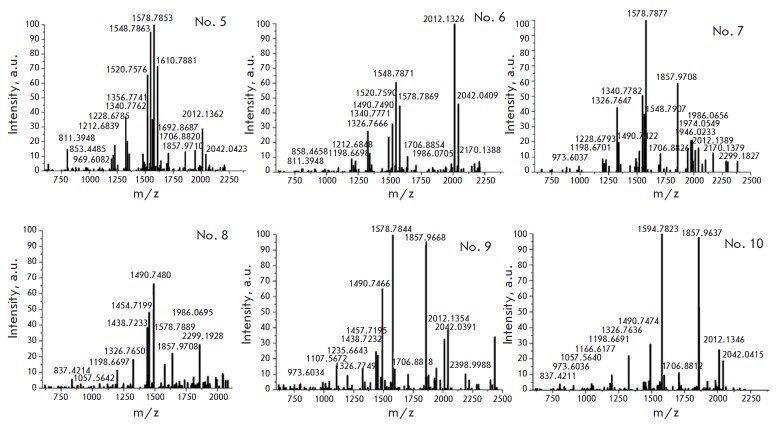
Mass spectrum of the 2D MEFs H1 zones

**Fig. 4:**
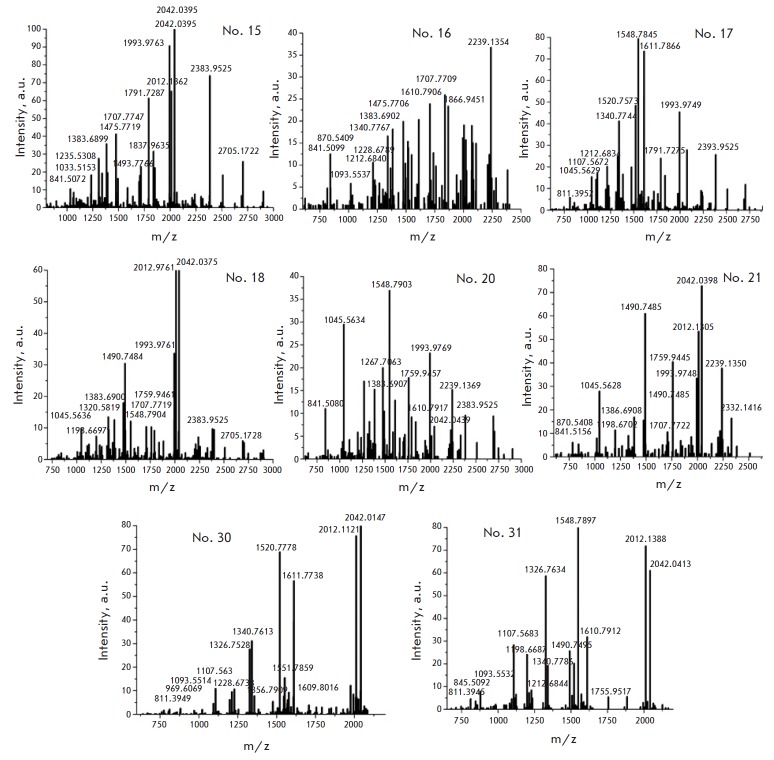
Mass spectrum of the 2D ES H1 zones

The objective of this study was to compare the PTMs of linker histones H1 from differentiated and pluripotent mouse stem cells. To separate the histone H1variants, we used a combination of AU-PAGE and SDS-PAGE, which is especially versatile for identifying charged acid-soluble proteins, including histones [7, 8, 23, 24]. *Figure 1* shows the results of 2-D electrophoretic separation of H1 from two types of differentiated cells (namely, spontaneously immortalized mouse embry onic fibroblasts (line NIH/3T3) and primary mouse embryonic fibroblasts (MEFs)) and from pluripotent stem cells (namely, mouse ES cells (line E14)). We identified H1 subtypes in NIH/3T3 cells (five fractions; *Fig. 1A*), MEFs (seven fractions; *Fig. 1B*), and ES cells (eight fractions; *Fig. 1C*). The remaining fractions were attributed to members of High Mobility Group family proteins and other nuclear proteins (*Table S1* [25]**)**. The results of the MS analysis of H1 are presented in *Table S2* [25] and *Fig. 2*, *Fig. 3*, *Fig. 4*.

**Fig. 5:**
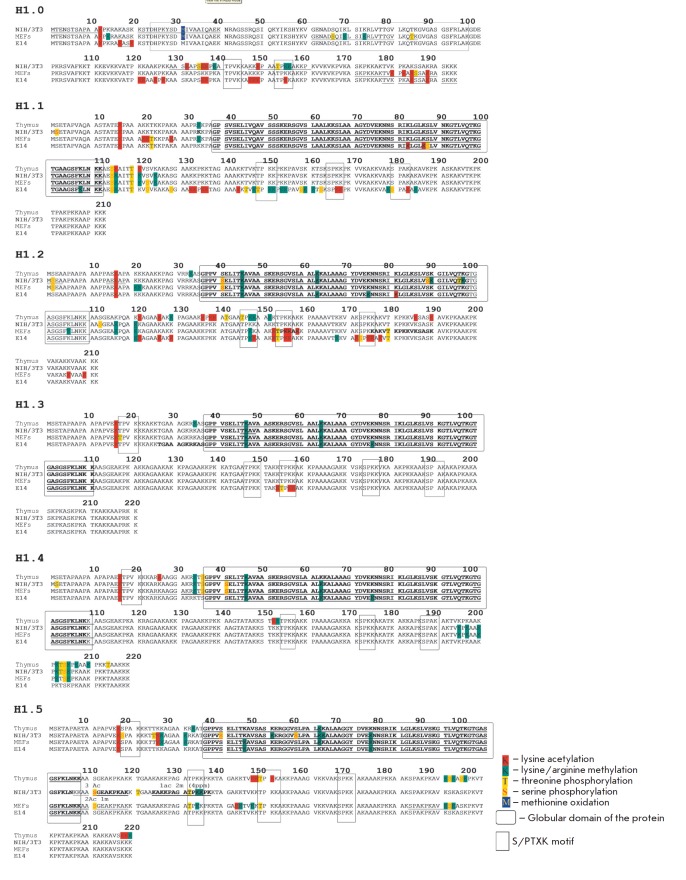
Potential post-translational modifications of H1 variants from NIH/3T3 cells, MEFs, and ES cells. The globular domain of H1 is shown with a rectangle with round edges. The S/PTXK region is shown with a rectangle

Six H1 isoforms (H1.0, H1.1, H1.2, H1.3, H1.4, and H1.5) were detected and analyzed. We identified PTMs of H1 from NIH/3T3, MEFs, and ES cells (*Table*), which were represented by acetylation, methylation, and phosphorylation. The results are summarized in *Fig. 5*, which additionally includes the previously identified PTMs of H1 from mouse thymus [7]. The data for the H1.0 mouse thymus variant were missing, so we relied on the data obtained for MEFs and NIH/3T3 cells.

## DISCUSSION

**Methylation**

H1 histones represent one of the main groups of nuclear proteins of chromatin that participate in the longitudinal compaction of replicated chromosome [24]. In chromatin of ES cells, there are 0.5 molecules of total H1 histone per nucleosome, which is twofold lower than in chromatin of differentiated cells [26]. Depletion of linker histone H1 in mice reduces chromatin compaction, global nucleosome spacing, and the overall levels of PTMs of some histones [26].

A comparative analysis of the H1 variants from NIH/3T3, MEFs, and ES cells revealed that the overall methylation of the H1.4 and H1.5 variants in ES cells was reduced compared to that in differentiated cells (*Fig. 5*). The identified methylation of H1 proteins in this region occurred at K34/K35, K63/65, and K73/75, depending on the H1 variant (*Table*).

**Table:** Potential modifications of the H1 histone variants from NIH/3T3, MEF, and ES cells identified by MALDI mass spectrometry. The modifications previously described in the literature are shown in bold

	H1 variant	modifications	Modification position
NIH/3T3	H1.0	Acetylation	**K12**, K132, K136, K137, K149
		Methylation	K139, K155, K156
		Phosphorylation	S135, T153
	H1.1	Acetylation	K17
		Methylation	K116, K121, K125
		Phosphorylation	S2, **S115**, T120
	H1.2	Acetylation	K17
		Methylation	**K46, K63**, K90, **K97, K117**, K121
		Phosphorylation	**S2**, S41, S89, T96, S113
	H1.3	Acetylation	K17
		Methylation	**K47, K64**
	H1.4	Acetylation	K17
		Methylation	**K34, K46, K63**, K195, K197, K200, **K202, K205**
		Phosphorylation	T18, S36, S41, T45
	H1.5	Acetylation	**K17**, K26, K12, K 180
		Methylation	**K27**, K31, K51, K62, **K63**, K74
		Phosphorylation	**S18**, T25, S40, S57, S111, T121, T132
MEFs	H1.0	Acetylation	**K12**, K180, K182, K184, K188
		Methylation	K14, K69, K73
		Phosphorylation	S66, T84, S185
	H1.1	Acetylation	**K17, K22**, K23, K29
		Methylation	**K35**, K116 , K121 , K125
		Phosphorylation	T24, **S115**, T120, S123
	H1.2	Acetylation	**K17**, K153, K156, K157, K159, K206, K210
		Methylation	**K21, K22, K46, K106, K117**, K121, **K148**
		Phosphorylation	**S2**, S41, **T154, T173**
	H1.3	Acetylation	K17
		Methylation	**K47, K64**
		Phosphorylation	T18
	H1.4	Acetylation	K17
		Methylation	**K34, K46, K63**, K195, K197, K200, **K202, K205**
		Phosphorylation	**S36, S41**, T45, **S204**
	H1.5	Acetylation	**K17**, K26, K143
		Methylation	**K27**, K31, **K45**, K62, K74, K134, K144, K147, K191, K193
		Phosphorylation	S111, T132, T149, S192
ES cells	H1.0	Acetylation	**K12**, K17, K20, K121, K122, K125, K127, K136, K137, K147, K148, K149, K155, K184, K188
	H1.1	Acetylation	**K17**, K83, **K87**, K133, K134, K136, K137, K144, K167, K168, K183
		Methylation	K108, K116, K148, K151, K152, K154, K155, K160, K161, K179, K185
		Phosphorylation	T24, S88, **S115**, T120, S123, S129, T146, T149, S159, S163, S180
	H1.2	Acetylation	**K17**, K81, **K122, K127**, K130, K149, K153, K156, K157, K172, K175, K176, K178
		Methylation	**K46, K63**, K75, K121, **K148, K168**
		Phosphorylation	**T154, S173**, T180
	H1.3	Acetylation	**K17**, K154, K157, K158
		Methylation	**K47, K64**, K75
		Phosphorylation	T155
	H1.4	Acetylation	K17
		Methylation	**K46, K63**, K75
	H1.5	Acetylation	K17
		Methylation	K45, K74

Many of the PTMs, such as meK63/64 for the H1.2-H1.4 variants, meK47 for H1.3, meK97 for H1.2, meK117 for H1.2, and meK27 for H1.5, have been previously reported [7, 8, 10-12]. Methylation at these positions is thought to protect the ε-amino groups of lysines by increasing histone affinity to DNA and facilitating their transition to a locally repressed chromatin state [7, 8]. Importantly, we identified methylation at K75 for the H1.2-H1.4 variants exclusively in ES cells (*Fig. 5, Table 2S* [25]). This PTM is located within the globular domain and may result in the protection of the ε-amino groups of the lysines in these cells.

Methylation of K108, K148, K151, K152 K154, K155, K160, K161, K179, and K185 in H1.1, as well as that of K168 in H1.2, has been identified exclusively in ES cells, whereas methylation of K202 and K204 in H1.4 may be limited to differentiated NIH3T3 cells and MEFs. Most of these PTMs are located within S/TPXK or (S/T) PXZ motifs near the phosphorylated serines and threonines of H1. The potential role of these modifications will be discussed in the Methyl/acetyl/phospho crosstalk section.

**Acetylation**

Our data demonstrated that the overall H1 acetylation level in ES cells had increased compared to that in differentiated cells (*Fig. 5*). As expected, we identified multiple acetylation sites in the N-terminal and globular domains of H1 (*Table*). In most cases, the exact biological role of these modifications remains unknown. One of the best studied acetylation sites is acK34-H1.4. The acK34-H1.4 is a hallmark of the promoters of the transcriptionally active gene and helps recruit the general transcription initiation complex TFIID to the promoters [27]. However, we have not identified this PTM in NIH/3T3, MEFs, and ES cells. We found methylation at this position of H1.4 in NIH/3T3 and MEFs but not in ES cells; the role of these modifications is not clear yet. Methylation protects the ε-amino groups of lysine, thus increasing histone affinity to DNA and facilitating the transition to a locally repressed chromatin state. Demethylation of K34-H1.4 in ES cells, on the other hand, may favor acetylation at this site and facilitate recruitment of the general transcription factor TFIID to the promoters.

AcK83 and acK87 of H1.1 and acK81 of H1.2 have been identified exclusively in ES cells. Reduction in the positive charge in this region due to acetylation of the amino group of lysine residues may destabilize H1- DNA interactions, resulting in the formation of a locally relaxed chromatin state.

The formation of open chromatin may also be facilitated by acetylation of lysine residues at the C-terminal regions of the H1.1-H1.3 variants. The reduced positive charge of the C-terminal domains of H1 proteins could weaken DNA/H1 interactions at the entry/exit regions of the core particle and prevent H1 interaction with regulatory chromatin proteins. Moreover, most of these C-terminal ES cell-specific acetylation and methylation sites of the H1.1-H1.3 variants are located within the S/ TPXK or (S/T) PXZ motifs near the phosphorylated serines and threonines. Their potential biological role and the mechanism of regulation of H1-DNA interaction mediated by acetylation/methylation of lysins within the S/TPXK or (S/T)PXZ motifs will be discussed in more detail in the Methyl/acetyl/phospho crosstalk section.

**Phosphorylation**

We identified several phosphorylation sites of H1: T24, S115, T120, and S123 of H1.1, S2, S41, T154, and T173 of H1.2 in both differentiated and ES cells. However, phosphorylation of S129, T146, T149, S159, S163, and S180 of H1.1; T180 of H1.2; and T155 of H1.3 were identified exclusively in ES cells, whereas S36 and S204 of H1.4 were not phosphorylated specifically in these cells (*Fig. 5, Table 2S* [25]). The identified phosphorylation sites are located mainly in the C-terminal portions of H1 variants, and some of these are located within the methyl/acetyl-phospho motifs (S/T)PXK and (S/T) PXZ, which are phosphorylated during mitosis, resulting in the modulation of chromatin states (*Fig. 5*) [15, 28-34]. It remains to be experimentally determined whether the observed phosphorylation at some sites and/or lack thereof at the other sites within H1 variants is functionally related to the maintenance of the pluripotent states of ES cells and/or the differentiation capacity of these cells.

Phosphorylation at S173 (H1.2) and S187 (H1.4) occurs during interphase and is necessary for chromatin relaxation and activation of transcription [15, 30-32]. Taking into account the fact that these serines lie within the methyl-phospho switch motifs, methylation of K172 of H1.2 in ES cells may promote phosphorylation of the adjacent S173. The pS173 may, in turn, promote acetylation of K172, leading to transcription activation.

**Methyl/acetyl/phospho crosstalk**

In addition to stand-alone PTMs of H1, we identified several conjoint PTMs, such as the following methylation/phosphorylation sites: meK148/pT149-H1.1 and meK179/pS180-H1.1 in ES cells, meK191/pS192-H1.5 in MEFs, which are located mainly in the C-terminal regions of the proteins (*Fig. 5*). Their structural organization resembles the methyl-phospho switch regions of core histones; one relevant example is the K9/S10 site in histone H3 [35-38]. The regulatory state of the K9/S10 site is characterized by a stable meK and dynamic phosphorylation of the S/T residue located next to K. Phosphorylation of S10 and S28 in H3 leads to acetylation at K9 and K27, respectively, resulting in transcription activation [39].

In addition, we also identified several other acetylation/phosphorylation sites, including acK17/pT18 in H1.4 and H1.5 from NIH/3T3 cells, acK17/pT18 in H1.3 from MEFs, acK23/pT24 in H1.1 from MEFs, acK184/ pS185 in H1.0 from MEFs, acK153/pT154 in H1.2 from MEFs and ES cells, acK154/pT155-H1.3 from ES cells, and acK172/pS173 in H1.2 from ES cells. These acetylation/phosphorylation regions are characteristic of both ES and differentiated cells. Their structural organization resembles that of the methyl-phospho switch regions, with the only exception that methylation changes to acetylation. It is possible that the mechanisms of methyl/acethyl-phospho region regulation of H1 are similar to those discussed above for the methyl-phospho switch regions of core histones [40-41]. In this scenario, acetylation of the lysines within the K(S/T) motif may lead to transcription activation in a similar fashion. This hypothesis, however, requires further experimental validation.

**Citrullination**

Citrullination of H1.2 to H1.4 at R54 promotes acquisition and maintenance of the pluripotent cell state [42]. Mechanistically, it displaces H1 from chromatin, promoting an open chromatin state. Citrullination is the replacement of arginine with citrulline. This change leads to the displacement of the peak of ERSGVSLAALK peptide at 0.9844 m/z in the mass spectra. We observed a “displacement” peak of low intensity in the region of 1131.64 m/z, but the determination accuracy is expressed as 9.8 ppm. When analyzing the modifications, we did not take into account peaks higher than 3.0 ppm. Therefore, we cannot clearly establish whether citrullination takes place in our H1.2–H1.4 ES samples. Additional studies and MS/MS mass spectrometry are needed to verify this assumption.

**Formylation**

Formylation of H1 variants was revealed in H1.2 at the K63-K85 and K97 positions in mouse tissues but not in cell lines [43]. We did not identify H1 formylation sites in H1 variants from the cells. The biological role of formylation is unknown, but it has been suggested that a specific enzyme can catalyze formylation during demethylation of lysines by amine oxidase LSD1 [44].

**Oxidation**

We identified the oxidation site for methionine at the M31 position for H1.0 of NIH/3T3 and MEFs but not in ES cells (*Table 2S* [25]). Oxidation of methionine produces MetO (methionine sulfoxide) [45]. The positions of M residues in proteins often contribute to the formation of the hydrophobic bonds between their sulfur atoms and rings of the aromatic residues of tryptophan, phenylalanine, or tyrosine [46]. These hydrophobic sulfur-ring bonds ensure the structural stability of proteins, which is approximately equal to that of an ionic salt bridge [46]. The interaction with M establishes the optimal positioning needed to ensure antioxidant protection of aromatic amino acids. Oxidation of methionine to MetO destroys this hydrophobic bond and may destroy the normal protein 3D folding. Oxidized proteins are characterized by increased surface hydrophobicity [47], which correlates with the age-related increase in the MetO content [45]. The absence of oxidation sites of H1 in ES cells is consistent with the unlimited self-renewal potential of these cells.

## CONCLUSIONS

In this study, we compared the PTMs of H1 from differentiated and pluripotent cells. We have shown that the total levels of methylation/acetylation of H1.3– H1.5 in ES cells are similar to those in differentiated cells; however, we have not found any significant differences between the nature and positions of the post-translational modifications in the H1.3-H1.5 proteins of ES and differentiated cells. In addition to reduced H1.0 expression levels in pluripotent cells [20], we have demonstrated that H1.0 and H1.2 are also characterized by an increased acetylation in ES cells (*Fig. 5*). The majority of acetylation sites in H1.0 and H1.2 from ES cells are located within the C-terminal domains of the proteins, namely in the 97–121 and 145–169 regions. These regions are present within the two known sub-domains of the C-terminal tail, which are involved in the stabilization of condensed chromatin [20, 48]. Reduction of the positive charge of the N- and C-terminal regions of H1 proteins could weaken the H1–DNA interaction at the entry/exit regions of the core particle and prevent H1 interaction with regulatory chromatin proteins such as HMGN and HMGB1/2 [49-50]. It is known that HMGB1/2-proteins are able to displace histone H1, thus facilitating nucleosome remodeling and modulating the accessibility of nucleosomal DNA to transcription factors or other sequence-specific proteins [51]. Displacement of H1 from the nucleosome should lead to the formation of an open chromatin structure, which is characteristic of stem cell chromatin.

Thus, an open structure of chromatin in pluripotent stem cells can be effected both by a reduction of the total level of H1 expression and by the presence of post-translational modifications in H1 proteins (H1.0, H1.2), which lead to disruption of their binding to DNA and, as a consequence, to the formation of chromatin with a looser structure. The biological role of the currently best known H1 modifications is not clear yet. Further studies are required to identify the functional roles of PTMs and to elucidate their crosstalk. This knowledge will contribute to a deeper understanding of the molecular processes that underlie the chromatin function in pluripotent cells.

## Abbreviations

MALDI-FT-ICR-MS: Fourier transform ion-cyclotron resonance mass spectrometry
PTM: post-translational modifications
ESC: embryonic stem cell
MEF: mouse embryonic fibroblast
AU-PAGE: acetic acid-urea polyacrylamide gel electrophoresis
SDS-PAGE: sodium dodecyl sulfate polyacrylamide gel electrophoresis
meK: lysine methylation
acK: lysine acetylation
pS/T: serine/threonine phosphorylation
MetO: methionine sulfoxide
